# Indents

**Published:** 1923-10

**Authors:** 


### INDENTS

**Brief Articles of Technical or Scientific Value will receive notice and comment. Contributions invited.**

---

"BITE-TAKING" AND "TRYING-IN." In taking a "bite" or "trying-in" teeth, always have the patient standing up in front of the operator, in a perfectly easy and natural position. A patient is more likely to "bite" correctly than when the head is leaning back in the head-rest of the chair. The center of the face, the slope of the teeth, and the "height" of the "bite" can also be much more accurately determined.—*British Dental Journal.*

A "DOUBLE-BARRELLED" METHOD OF TAKING A BITE IN AN

---

EDENTULOUS MOUTH. It is usually found that the "bite" is too keen at the ends of the jaw—often the wax is entirely bitten through—so that the hard bite-plates come in contact with each other at the ends. It is, of course, necessary and desirable that there be an *even* thickness all around the biting surface of homogeneously soft wax. To accomplish this, remove the bite-plates from the mouth, mount them on an articulator frame to the height of the bite given. Remove the wax, when it will be seen how much to cut down, or build up the lower bite-plate in order to get an even distance all around between the bite-plates. Having done this, take another "bite" with freshly-softened wax, when it will be found that the bite pressure will be evenly distributed all around.—*British Dental Journal.*

---

FULL CAST CROWN. To make a full cast crown, prepare the tooth by the removal of all enamel, or at least sufficient to produce a cone-shape with the elimination of all undercuts. If it is a vital tooth, paint this prepared stump with saturate silver nitrate solution and precipitate with powdered silver (conveniently applied as powdered amalgam on a pellet of cotton) or with eugenol. Fit a

Barrack twenty-two or twenty-four carat thirty gauge band snugly to the prepared stump, slitting it around the occlusal periphery and bending it into actual contact with the sides and across the top of the stump. Take an impression and bite over this; pour model of investment material or with plaster after filling the crown band with wet soft paper or in some other way providing for its ready removal from the cast; build the crown up with wax and carve to correct contour; invest and cast. Another very good method of making a full cast crown either with or without a shoulder is: After the root is prepared properly, fit a copper band and take a correct modelling compound impression of the prepared stump, allowing the compound to chill thoroughly before removal. Surround this impression with a box of stiff paper or wax and pack with amalgam, making an amalgam model of the prepared root. This should be set in place in an impression and bite that have been previously procured from the mouth. This amalgam model should be so shaped and waxed as to be removed readily from the plaster cast for the purpose of swaging. Now burnish or swage twenty-two or twenty-four gauge pure gold to the amalgam model; build up with wax; carve to correct contour; invest; cast; replace upon the amalgam model and swage. Another nice way is to oil the amalgam model and dip it into the melted wax, repeating same until the wax is about thirty gauge. Place a sprue; remove and cast with pure gold. Swage this upon the amalgam model, build to correct contour with wax and recast with twenty-two carat or whatever gold alloy you wish to use. Re-swage upon the amalgam model and polish.—*Dental Digest.*

